# Interaction of gut microbiota with the tumor microenvironment: A new strategy for antitumor treatment and traditional Chinese medicine in colorectal cancer

**DOI:** 10.3389/fmolb.2023.1140325

**Published:** 2023-03-06

**Authors:** Tingting Li, Liang Han, Simin Ma, Weiji Lin, Xin Ba, Jiahui Yan, Ying Huang, Shenghao Tu, Kai Qin

**Affiliations:** ^1^ Department of Integrated Traditional Chinese and Western Medicine, Tongji Hospital, Tongji Medical College, Huazhong University of Science and Technology, Wuhan, China; ^2^ Department of Nosocomial Infection Management, Tongji Hospital, Tongji Medical College, Huazhong University of Science and Technology, Wuhan, China

**Keywords:** colorectal cancer, tumor microenvironment, gut microbiota, traditional Chinese medicine, treatment

## Abstract

Colorectal cancer (CRC) is one of the most common malignancies worldwide and the second leading cause of cancer-related death. In recent years, the relationship between gut microbiota and CRC has attracted increasing attention from researchers. Studies reported that changes in the composition of gut microbiota, such as increase in the number of *Fusobacterium nucleatum* and *Helicobacter hepaticus*, impair the immune surveillance by affecting the intestinal mucosal immunity and increase the risk of tumor initiation and progression. The tumor microenvironment is the soil for tumor survival. Close contacts between gut microbiota and the tumor microenvironment may directly affect the progression of tumors and efficacy of antitumor drugs, thus influencing the prognosis of patients with CRC. Recently, many studies have shown that traditional Chinese medicine can safely and effectively improve the efficacy of antitumor drugs, potentially through remodeling of the tumor microenvironment by regulated gut microbiota. This article describes the effect of gut microbiota on the tumor microenvironment and possible mechanisms concerning the initiation and progression of CRC, and summarizes the potential role of traditional Chinese medicine.

## 1 Introduction

Colorectal cancer (CRC) is the third most common type of cancer worldwide (Baidoun et al., 2021; Schmitt and Greten, 2021). Its annual global incidence rate is nearly 1 million cases, and the annual mortality rate associated with this disease is at least 600,000 cases (Sui et al., 2020), accounting for approximately 10% of cancer-related deaths worldwide (Ren et al., 2021). Genetics, environment, lifestyle, and increasing age are the main risk factors for CRC (Lucas et al., 2017). In recent years, there has been increasing attention on the relationship between the gut microbiome and CRC (Jain et al., 2021). The human gut microbiome includes at least trillions of microbes that play a vital role in human health and disease (Hayase and Jenq, 2021). The components of the gut microbiome (e.g., *Escherichia coli*, *Bacteroides fragilis*, *Fusobacterium nucleatum*) may disrupt immune surveillance by affecting intestinal mucosal immunity, thus promoting the development and progression of CRC (Clay et al., 2022). The tumor microenvironment (TME) consists of immune cells [e.g., T, B, and natural killer (NK) cells] and a variety of bone marrow cell populations [e.g., granulocytes, monocytes, macrophages, and dendritic cells (DC)], abnormal vasculature, and immunosuppressive cytokines, which exert crucial function in tumor immune tolerance (Qiu et al., 2020). The intimate contacts between gut microbiota and the TME may directly induce TME reprogramming and exhibit a profound impact on tumor immunity. Consequently, these effects are closely related to the tumor development, the efficacy of antitumor drugs, and the prognosis of patients with CRC (Hayase and Jenq, 2021). Traditional Chinese medicine (TCM) has a history of thousands of years in the prevention and treatment of diseases. Data have shown that TCM significantly improves the sensitivity to chemotherapy drugs and remarkably alleviates cancer-related adverse reactions. TCM offers unique advantages in prolonging the survival of patients with cancer and improving their life quality (Zhang et al., 2020). As an alternative therapy, TCM can safely and effectively improve the efficacy of antitumor drugs in the treatment of CRC, potentially through remodeling of TME by regulated gut microbiota (Qi et al., 2015). This article describes the impact of intestinal flora and its metabolites on the TME and possible mechanisms affecting the occurrence and progression of CRC. Besides, the potential role of TCM in this process is summarized.

## 2 Relationship between the gut microbiome and intestinal mucosal immunity

The intestine is the largest digestive organ of the human body with various important functions, including digestion and absorption of food, regulation of metabolism, transmission of information, *etc.,* (McDermott and Huffnagle, 2014). During body development, the gut microbiome maintains a mutually beneficial symbiotic relationship with the intestinal microenvironment. The intestine provides nutrients and an environment for the growth of intestinal flora. Intestinal commensal bacteria assist in the digestion and absorption of substances by reducing intestinal permeability and increasing epithelial defense mechanisms to form a mucosal barrier. Moreover, they provide beneficial nutrients [e.g., vitamin D, short-chain fatty acids (SCFA), *etc.*] to support the maturation of the intestinal immune system (Topping and Clifton, 2001; Shi et al., 2017). Due to the unique luminal structure of the intestine, the gut is constantly exposed to the gut microbiome and other various antigens. Hence, the body develops a unique immune system, which is a mature intestinal mucosal immune system composed of intestinal epithelial cells, Peyer plaques, isolated lymphatic follicles, mesenteric lymph nodes, *etc.*, to maintain intestinal homeostasis (Shi et al., 2017). In addition, intestinal flora plays an important role in promoting the formation of healthy and mature intestinal mucosal immunity and maintaining intestinal homeostasis. In early life, proper colonization of the gut flora results in pathogen-associated molecular patterns stimulating of pattern-recognition receptors expressed on intestinal mucosal epithelial cells or immune cells. This stimulation subsequently induces the maturation of intestinal mucosa-associated lymphoid tissue (Maynard et al., 2012). However, in certain cases of intestinal flora deficiency, the intestinal immune system is compromised. This compromise is manifested as incomplete development of intestinal mucosa-associated lymphoid tissue, reduction in the number of immune cells (e.g., plasma cells, lymphocytes) and cytokines, and downregulation of surface pattern-recognition receptor expression. In turn, these effects lead to the occurrence of certain intestinal diseases, including ulcerative colitis, Crohn’s disease, and CRC (Sommer and Bäckhed, 2013; Takiishi et al., 2017). The interaction between intestinal flora and the intestinal immune system makes it possible to improve local immune system function by regulating the composition of the intestinal flora, reshape the intestinal microenvironment, and treat or alleviate disease.

## 3 Effects of gut microbiota on the tumor inflammatory microenvironment

**FIGURE 1:**
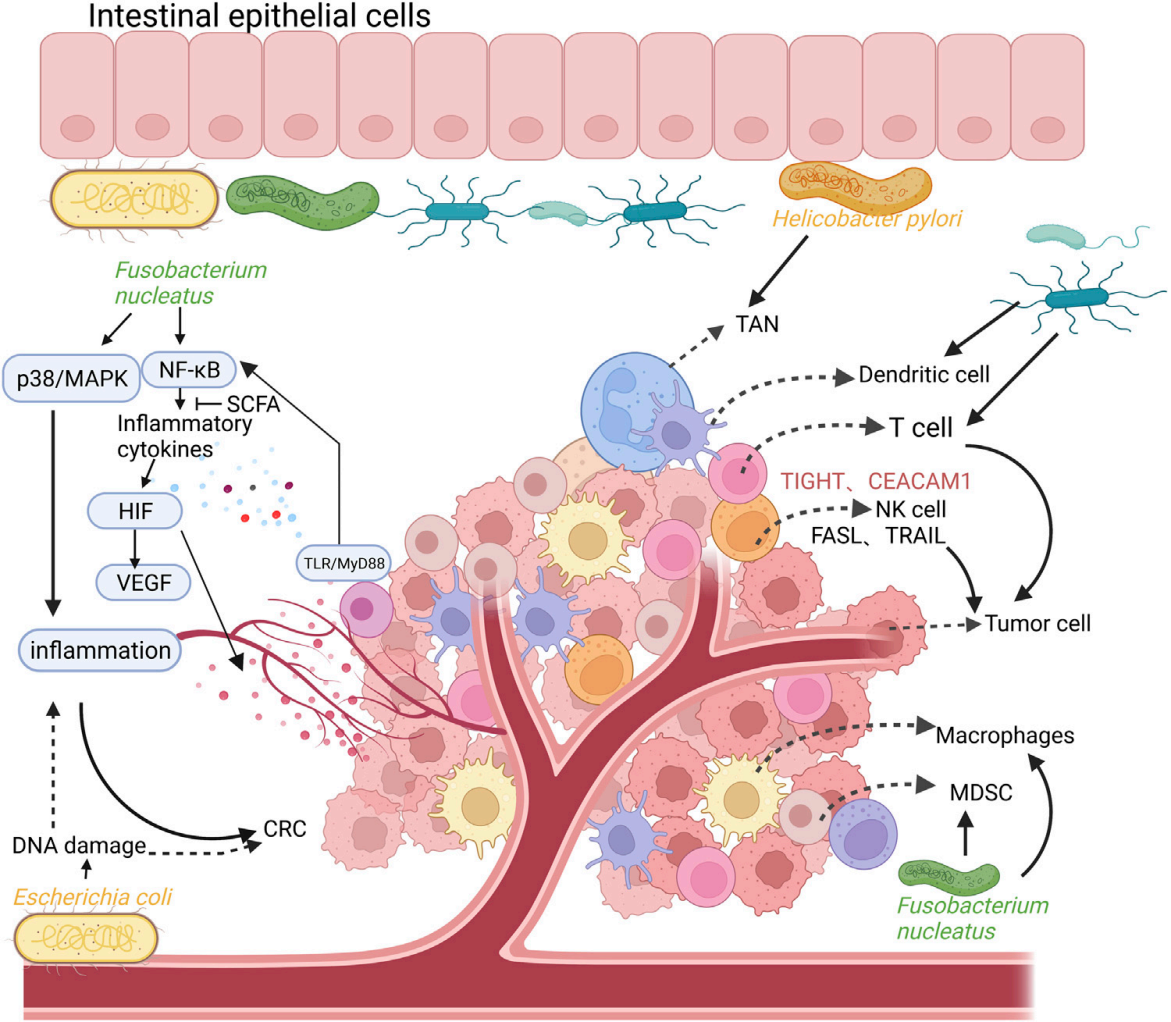
The relationship between intestinal flora, inflammation, colorectal cancer (CRC), and the composition of the tumor immune microenvironment. Intestinal commensal bacteria (such as *Fusobacterium nucleatum*) promote the activation of NF-κB and the expression of pro-inflammatory cytokines through TLR/MyD88 signaling on tumor-infiltrating cells, which can directly regulate the HIF pathway and promote the expression of VEGF, leading to angiogenesis in tumor tissues. In addition, *Fusobacterium nucleatum* can also promote p38 or MAPK signal-mediated pro-inflammatory responses, promoting the formation of CRC. Polyketide synthase-expressing (pks+) *Escherichia coli* can aggravate colon inflammation and malignant cell transformation by destroying the DNA of colon cells. The tumor immune microenvironment is mainly composed of T cells, tumor-associated macrophages (TAM), immunosuppressive myeloid-derived suppressor cells (MDSC), dendritic cells (DC), tumor-associated Neutrophils (TAN), NK cells. Created with BioRender.com.

Inflammation is considered a key driver for the development of CRC, and it is thought that at least 20% of cancer cases are a direct consequence of the chronic inflammatory process. Patients with inflammatory bowel disease, including ulcerative colitis and Crohn’s disease, are at a higher risk of CRC compared with healthy individuals (Eaden et al., 2001; Canavan et al., 2006; Farraye et al., 2010). Under normal circumstances, the intestinal mucosal barrier consists of a monolayer of intestinal epithelial cells through tight junctions, and the intestinal mucosal barrier isolates the gut microbiota from immune cells (Lee et al., 2018). However, in a state of chronic inflammation, the intestinal mucosal barrier is destroyed and its permeability is altered, leading to the invasion of the gut by commensal microorganisms and pathogenic microorganisms (Grivennikov et al., 2012). Some bacteria accumulate and anchor themselves in colonic epithelium to disturb the epithelial cytoskeleton and destroy the structure of the epithelial cell junction. On intestinal epithelial cells, for instance, invasion by enterotoxigenic *B. fragilis* cleaves E-cadherin, further disrupting the colonic mucosal barrier (Wu et al., 2007). The invading commensal bacteria interact with the toll-like receptor (TLR) on tumor-infiltrating bone marrow cells. Subsequently, activation of the transcription factor nuclear factor kappa-B (NF-κB) was promoted by the MyD88-dependent signaling cascade. This process induces the expression of genes encoding pro-inflammatory cytokines and chemokines (Zhao et al., 2021). The expression of inflammatory cytokines can directly regulate the hypoxia inducible factor (HIF) pathway, elevate the levels of vascular endothelial growth factor (VEGF) and promote angiogenesis in tumor tissues (Thornton et al., 2000; Pouysségur et al., 2006; Westra et al., 2007). VEGF-mediated angiogenesis plays a key role in the progression of human tumors (i.e., from adenoma formation to the development of non-invasive CRC). As a key regulator of tumor angiogenesis, VEGF accelerate the occurrence and progression of CRC *via* promoting the proliferation, migration, and differentiation of endothelial cells (Carmeliet, 2005). Moreover, commensal bacteria also upregulate the level of interleukin 17C (IL-17C) in intestinal epithelial cells through TLR/MyD88-dependent signaling. This induces B-cell CLL/lymphoma 2 (BCL2) expression in intestinal epithelial cells, which promotes tumorigenesis (Song et al., 2014; Cheng et al., 2020). Inflammation also results in DNA damage in colonic epithelial cells by increasing the release of reactive oxygen species (ROS) and nitrogen substances by innate immune cells (e.g., macrophages and neutrophils) into the tissue microenvironment. These effects initiate the development of malignancy during chronic intestinal inflammation (Cheng et al., 2020). In a mouse model of CRC, it was shown that *F. nucleatum* produced a pro-inflammatory environment that promoted the formation of CRC by activating the NF-κB pathway and recruiting tumor-infiltrating immune cells (Kostic et al., 2013). Invasion by *F. nucleatum* promotes p38 or mitogen-activated protein kinase (MAPK) signaling-mediated pro-inflammatory responses in HEK293T cells and mediates ROS production in Caco-2 cell lines; both processes are involved in the development of early CRC (Tang et al., 2016; Hanus et al., 2021). Moreover, in Apc^Min/+^ mice, *Peptostreptococcus anaerobius* extensively induced the expression of pro-inflammatory cytokines, triggered a pro-inflammatory immune microenvironment, and led to the recruitment of a series of tumor-infiltrating immune cells, especially immunosuppressive myeloid-derived suppressor cells (MDSC) and tumor-associated macrophages (TAM). These effects promoted tumor progression (Long et al., 2019). Polyketide synthase-expressing (pks^+^) *E. coli* can aggravate colon inflammation and malignant cell transformation by destroying the DNA of colon cells (Hanus et al., 2021) (Figure 1).

## 4 Effects of gut microbiota on the tumor immune microenvironment

### 4.1 T cells

Studies have shown that innate immunity and T cell-mediated adaptive immunity affect the evolution and development of tumors all the way (Wang Y et al., 2021). The interaction of specific gut microbiota with the TME contributes to antitumor effects. It was found that certain gut microbiota enhanced the expression of major histocompatibility complex (MHC) class I in DC, followed by activation of interferon gamma (IFN-γ)^+^CD8^+^ T cells, and increased the number of (IFN-γ)^+^CD8^+^ tumor-infiltrating lymphocytes in the TME to inhibit the growth of CRC (Tanoue et al., 2019). Invasion of the body by immunogenic gut microorganisms promotes follicular T helper (Tfh)-related antitumor immunity in the colon (Tanoue et al., 2019). For example, in mouse models of CRC, colonization by *Helicobacter hepaticus* induced an increase in the number of colonic CD4 Tfh cells and promoted the maturation of tumor-adjacent tertiary lymphoid structures, increased cytotoxic lymphocyte infiltration in tumor, and inhibited tumor growth (Overacre-Delgoffe et al., 2021). In addition, invasion by these intestinal microorganisms promotes the differentiation of CD8^+^ T cells into cytotoxic T-lymphocytes, which produce IFN-γ, tumor necrosis factor alpha (TNF-α), and Fas ligand to kill tumor cells. Furthermore, the presence of some gut microbes exerts immunosuppressive regulatory effects on the TME. Mima et al. (2015) found that the number of *F. nucleatum* in CRC tissue was inversely correlated with CD3^+^ T cell density. *Fusobacterium nucleatum* can inhibit the response of human T cells to mitogen and antigens, block T cells in the G1 phase of the cell cycle, inhibit T cell proliferation, promote their apoptosis (Shenker and Datar, 1995). In CRC, *F. nucleatum* induce programmed cell 1 death ligand 1 (PD-L1) expression by activating the stimulator of interferon genes (STING) signaling. Interestingly, *F. nucleatum* increased the accumulation of interferon-gamma (IFN-γ)^+^CD8^+^ tumor-infiltrating lymphocytes (TILs) during treatment with PD-L1 blockade, thereby augmenting tumor sensitivity to PD-L1 blockade and prolonging the survival of mice with CRC (Gao et al., 2021). In addition, *F. nucleatum* binds to and activates T cell immunoreceptor with Ig and ITIM domains (TIGIT) and CEA cell adhesion molecule 1(CEACAM1) expressed by T and NK cells, directly blocking their ability to kill tumor cells and suppressing antitumor immunity (Gur et al., 2015; Bashir et al., 2016).

### 4.2 TAM

Macrophages are essential innate immune cells for maintaining system homeostasis and one of the most important types of immune cells in the colonic TME. These cells interact with tumor cells through exosomes or secreted cytokines to promote the proliferative, migratory, and invasive abilities of tumor cells. Moreover, in some cases, macrophages can stimulate antitumor immunity or directly kill tumor cells (Wang H et al., 2021). Macrophages exhibit high plasticity and can polarize into either the M1 or M2 phenotype. M2 macrophages are one of the main cells that shape the tumor immunosuppressive microenvironment. It can produce high levels of ROS, leading to DNA damage and genomic instability, which promote tumor invasion and metastasis (Schreiber et al., 2011). Specific gut microbes can influence tumorigenesis and progression through TAM in the TME. Some microbiota stimulate macrophages to increase c-Jun phosphorylation and accelerate the proliferation of CRC cells (Li et al., 2012). *Fusobacterium nucleatum* induces the expression of tumor-derived C-C motif chemokine ligand 20 (CCL20), promotes the recruitment of macrophages and MDSC in the TME, and transforms macrophages to the M2 type. These effects result in the formation of an immunosuppressive microenvironment conducive to tumor progression and promote the metastasis of CRC (Xu et al., 2021). *Enterococcus faecalis* promotes tumorigenesis through a macrophage-induced bystander effect, and induces colonic macrophage polarization to the M1 type, which in turn induces aneuploidy and chromosomal instability in colon cancer epithelial cells (Wang and Huycke, 2007). Vancomycin-induced dysbiosis leads to the proliferation of colonic epithelial cluster cells and promotes the production of IL-25 by these cells. Subsequently, IL-25 activates M2 macrophages in the TME and increases the secretion of C-X-C motif chemokine ligand 10 (CXCL10), which promotes the infiltration of CD8^+^ T cells in the TME and tumor development (Li Q. et al., 2019). TAM induce epithelial–mesenchymal transition *via* the signal transducer and activator of transcription 3/miR-506-3p/forkhead box Q1(STAT3/miR-506-3p/FOXQ1) axis to support the growth and metastasis of CRC. In addition, they may serve as prognostic markers in patients with CRC (Wei C et al., 2019). The intestinal flora stimulates the secretion of the metastasis-related secretory protein cathepsin K, which binds to toll-like receptor 4 (TLR4) and induces the M2 polarization of TAM through the mechanistic target of rapamycin kinase-dependent (MTOR-dependent) pathway. This process has been associated with the metastasis of CRC (Li R. et al., 2019).

### 4.3 MDSC

MDSC are tumor-permissive myeloid cells with potent immunosuppressive activity. They are also an important component of the TME (Gordon, 2003). These cells inhibit innate and adaptive immunity, and gut microbiota-induced MDSC often exert immunosuppressive effects on the TME (Gabrilovich et al., 2012; Raber et al., 2014). Studies have shown that *F. nucleatum* can promote the production of MDSC in the TME, and increase the expression levels of arginase 1 (ARG1) and inducible nitric oxide synthase (iNOS) in these cells. These effects subsequently lead to significantly inhibited activity of T cell and promote intestinal tumorigenesis (Kostic et al., 2013). Clearance of *F. nucleatum* reduced the number of MDSC, remodeled the TME, and prolonged the survival of mice with CRC. In addition, enterotoxigenic *B. fragilis* recruited MDSC into the colon TME of mice *via* IL-17. Through interaction with IL-17 receptors, the bacterium indirectly induced intestinal epithelial cell ectopia to produce chemokines and growth factors. *Bacteroides fragilis* also induced the expression of submucosal IL-17 and promoted IL-17-mediated colonic inflammation. IL-17 and transformed colonic epithelial cells promote tumor development by inhibiting immune effector cells and activating the STAT3 signaling pathway, as well as the secretion of pro-angiogenic mediators, matrix metalloproteinase 9 (MMP9), and VEGF (Thiele Orberg et al., 2017).

### 4.4 DC

As a main participant in the immune surveillance of tumors, DC have a strong antigen presentation function, stimulate the activation and proliferation of naïve T cells, initiate specific immunity (Böttcher et al., 2018; Wculek et al., 2019; Yang et al., 2021). Intestinal microbiome can mediate antitumor immunity by regulating DC function and status in TME. Shi et al. (2020) reported that bifidobacteria can migrate into tumor tissue, activate the IFN gene stimulating factor (STING) immune signaling pathway in DC, and re-expose tumor cells for recognition by the immune system. Oral administration of *Bifidobacterium* can restore the antitumor efficacy of PD-L1-specific antibody therapy (checkpoint blockade) by enhancing the maturation of DC and increasing the activation and accumulation of CD8^+^ T cells in the TME (Sivan et al., 2015). *Bacteroides fragilis* enhances the antitumor effects of cytotoxic T-lymphocyte associated protein 4 (CTLA4) blockade by triggering the maturation of DC and stimulating the IL-12-dependent T helper 1 (Th1) cell immune response. In addition, *B. fragilis* can stimulate migrating DC to activate Tfh and promote the antitumor effects of chemotherapy and immunotherapy (Vétizou et al., 2015).

### 4.5 Tumor-associated neutrophils (TAN)

TAN are an important component of the tumor-infiltrating cell population, with strong chemotaxis and phagocytosis functions that are closely related to tumor occurrence and metastasis (Huang et al., 2019; Triner et al., 2019). MMPs secreted by neutrophils can inhibit the activity of T cells and mediate tumor immunity. In a model of colitis-associated CRC, anti-neutrophil antibodies reduced tumor size and neutrophil infiltration in the colon, as well as MMP9 mRNA expression. These findings indicated that TAN promote tumor development (Shang et al., 2012). It has been found that the presence of *Helicobacter pylori* in the intestine can increase the levels of nitric oxide (NO) and TNF-α production by neutrophils in the TME, as well as activate the NF-κB signaling pathway, leading to the occurrence of CRC. In addition, neutrophils can receive emergency signals from cytokines released in the TME, which enter the blood circulation in large quantities and accelerate metastasis (Erdman et al., 2009).

### 4.6 NK cells

NK cells play a role in both innate and acquired immunity, recognizing molecules induced on the cell surface by stress signals and viral infections. In the gut, NK cells are dispersed in the epithelium or stroma, in close contact with the gut microbiome and its components. In addition, they interact with various cells (e.g., macrophages and DC) in the intestine to develop an effective immune response that helps maintain intestinal immune homeostasis (Poggi et al., 2019). NK cells can trigger targeted cell death by releasing lysed particles, such as perforin and granzyme. They can also induce apoptosis and promote antitumor immunity by binding TNF-related apoptosis inducing ligand and Fas ligand to receptors on colorectal tumor cells (Qiu et al., 2020). However, the intestinal flora can act on NK cells, affect their biological behavior, and inhibit their antitumor activity. Studies have demonstrated that *F. nucleatum* can interact with the inhibitory receptor TIGIT on the surface of NK cells, inhibit T cell activation, and block the NK cell-mediated cytotoxic effect on colon tumor cells (Gur et al., 2015). NK cells interact with gut microbes and influence adaptive T cell-mediated immune responses by acting on specialized antigen-presenting cells (APCs), such as DC (Poggi et al., 2019). Studies have shown that *Lactobacillus plantarum* produces protective immunity through an increase in infiltration by CD8^+^ T cells and NK cells, as well as an increase in IFN-γ production in the TME. In addition, *L. plantarum* can promote IL-22 production through the stimulation of NK cells and defend against intestinal epithelial barrier damage induced by enterotoxin-producing *E. coli* (Suzuki, 2020; Yang et al., 2021).

## 5 Effects of metabolites of gut microbiota on the TME

### 5.1 SCFA

Some metabolites of the gut microbiota, such as SCFA (e.g., butyrate, acetate, and propionate), also interact with the TME and play an important role in maintaining intestinal homeostasis (Yang et al., 2020; Sepich-Poore et al., 2021). Some bacteria, such as *Hodmanella bismorphoformis*, exhibit antitumor activity by increasing SCFA production in adenomas and inhibiting histone deacetylase. Low levels of these bacteria were detected in CRC. Smith et al. (2013) reported that SCFA help maintain intestinal immune homeostasis and regulate intestinal barrier function. Moreover, it interacts with the regulatory T (Treg) cell receptor G protein-coupled receptor 43 (GPR43) in the intestine, and upregulates the gene expression of FOXP3 and IL-10 in Treg cells of germ-free mice, consequently inhibiting effector CD4^+^ T cells and alleviating colitis. In addition, SCFA can also exert anti-inflammatory effects by inhibiting the activity of histone deacetylase in neutrophils, reducing the production of TNF-α and nitric oxide, and inhibiting NF-κB signaling. Butyrate is the most abundant SCFA in the intestinal flora and a crucial source of energy for colon cells. It has the ability to inhibit angiogenesis and reduce the expression of proangiogenic factors. Furthermore, it can increase the polarization of M2 macrophages, enhance the cytotoxic function of CD8^+^ T cells, block the production of DC in bone marrow by inhibiting histone deacetylase, and enhance the immunosuppressive function of Treg cells (Davie, 2003; Arpaia et al., 2013; Furusawa et al., 2013; Zagato et al., 2020). In addition, butyrate can exert antitumor effects by inhibiting oncogenic pathways, such as WNT and NF-κB (Zuo et al., 2013). It also induces an anti-inflammatory host response in bone marrow cells *via* GPR109a in the colonic epithelium, mediating the production of regulatory T cells. This inhibits the occurrence of colitis and colon cancer (Singh et al., 2014).

### 5.2 Bile acids

Studies have revealed that bile acids, as intestinal microbiogenic metabolites, exert immunosuppressive effects. The production of bile acids is closely related to a high-fat diet, which can lead to a marked increase in the secretion of primary bile acids by the intestine. The intestinal flora can convert primary bile acids into secondary bile acids, which induce the expression of FOXP3, increase the number of Treg cells, and promote immune escape (Campbell et al., 2020). In addition, deoxycholic acid and lithocholic acid may cause DNA damage by increasing ROS production, which in turn leads to cellular senescence, chronic inflammation, and CRC (Boleij and Tjalsma, 2012; Hibberd et al., 2017).

### 5.3 Inosine

Inosine binds to the adenosine A2a receptor in T cells in the presence of IFN-γ, promotes the differentiation of Th1 cells, and enhances the efficacy of immune checkpoint inhibitors (e.g., anti-CTLA4 and anti-PD-L1) (Mager et al., 2020; Yang et al., 2021). *Bifidobacterium pseudolongum* enterica can enhance the response to immunotherapy by producing the metabolite inosine (Mager et al., 2020).

### 5.4 Polyamines

**FIGURE 2:**
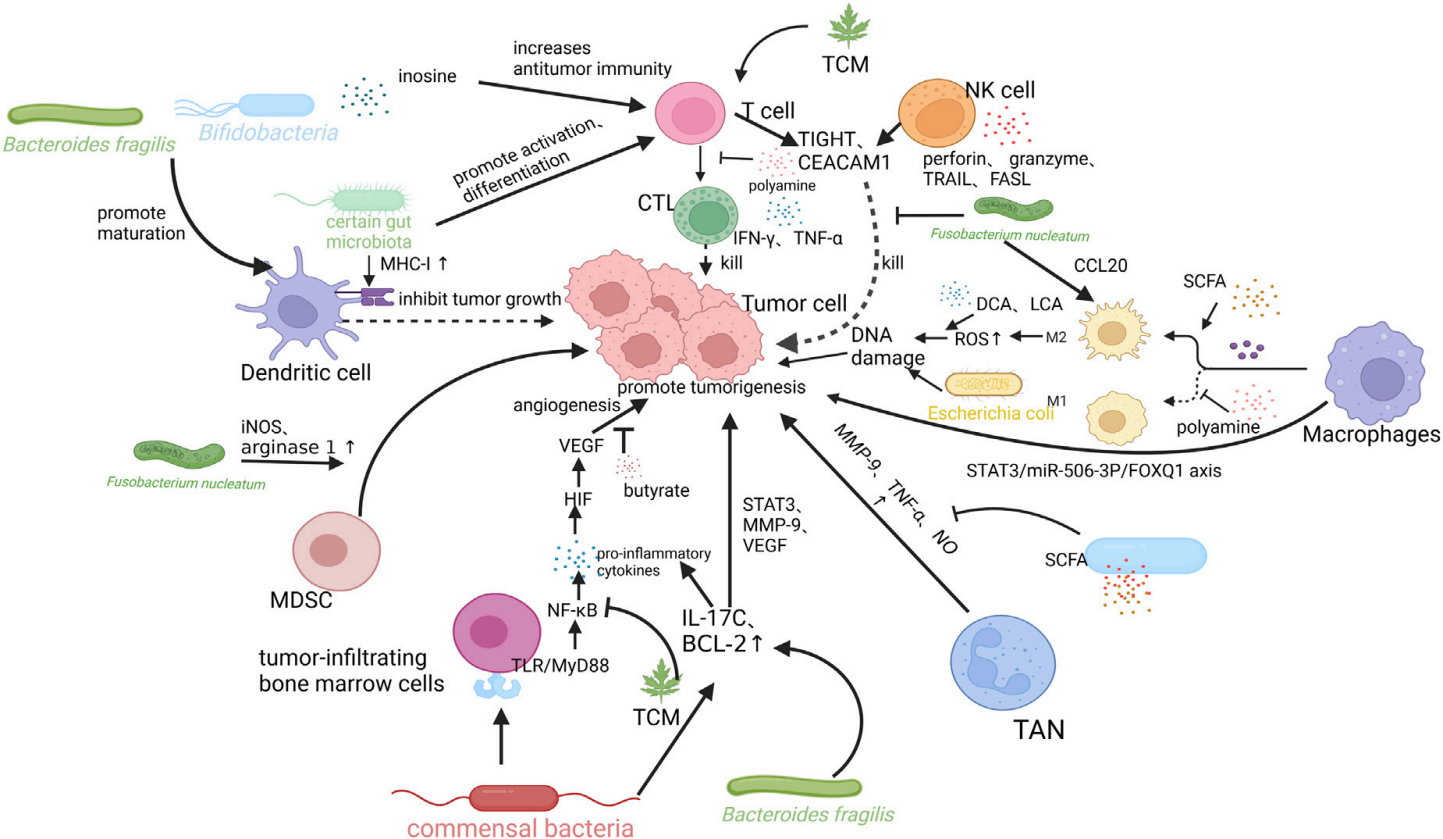
Mechanisms of intestinal flora and its metabolites, tumor microenvironment (TME), and traditional Chinese medicine (TCM) interactions. *Bifidobacteria* and *Bacteroides fragiligis* can promote the maturation of DCs and promote anti-tumor immunity. Certain gut microbiota can promote the expression of MHC-I in DCs, promote T cell differentiation into CTL, produce IFN-γ, TNF-α kill tumor cells. *Fusobacterium nucleatum* can recruit MDSCs in TME and increase the expression levels of arginase 1 (ARG1) and inducible nitric oxide synthase (iNOS) in these cells, which in turn inhibits the activity of T cells and promote intestinal tumorigenesis. *Bacteroides fragilis* also induces submucosal IL-17 expression and promotes IL-17-mediated colonic inflammation. SCFA can also exert anti-inflammatory effects by reducing the production of TNF-α and nitric oxide (NO), and inhibiting NF-κB signaling. It can also mediate antitumor immunity by increasing the polarization of M2 macrophages. M2 macrophages cause DNA damage by producing high levels of ROS, promoting tumor invasion and metastasis. Deoxycholic acid (DCA) and lithocholic acid (LCA) may promote CRC in this way. *Fusobacterium nucleatum* induces the expression of CCL20 and transforms macrophages to the M2 type. Tumor-associated macrophages (TAMs) can also induce EMT *via* STAT3/miR-506-3p/FOXQ1 axis to support the growth and metastasis of CRC. NK cells can trigger targeted cell death by releasing lysed particles, such as perforin and granzyme. They can also induce apoptosis and promote antitumor immunity by binding TNF-related apoptosis inducing ligand and Fas ligand to receptors on colorectal tumor cells. *Fusobacterium nucleatum* binds to and activates TIGIT and CEACAM1 expressed by T and NK cells, directly blocking their ability to kill tumor cells and suppressing anti-tumor immunity. TCM can exert antitumor effects by influencing the activation of T cells, inhibiting inflammatory pathways, and regulating the composition of intestinal flora. Created with BioRender.com.

Polyamines (e.g., putrescine, spermidine, spermine, and cadaverine) are derived from the endogenous synthesis of amino acids, diet, and intestinal microbiota metabolism. They are involved in angiogenesis, immune response, intestinal barrier function, and epithelial renewal. Polyamines are essential for the activation of T and B cells (Cervelli et al., 2014; Coni et al., 2019). Changes in polyamine levels are associated with the development of cancer (Ramos-Molina et al., 2019). Fecal metabolomic analysis showed increased levels of polyamines in patients with CRC, which may be attributed to uptake pathways and enzyme synthesis (Milovic and Turchanowa, 2003; Yang et al., 2019). The formation of biofilms in colon cancer increases the levels of polyamine metabolites (Johnson et al., 2015). In CRC, the increase in polyamine levels reduces the expression of adhesion molecules (e.g., CD44) and the production of cytokines (e.g., IFN-γ and TNF-α), resulting in immunosuppression of the TME (Zhang et al., 1997; Haskó et al., 2000; Soda, 2011; Tsujinaka et al., 2011; Hesterberg et al., 2018). In addition, the study also found that polyamines can alter the chromatin structure and prevent inflammatory gene transcription to inhibit macrophage transformation to the M1 type (Hardbower et al., 2017; Latour et al., 2020) (Figure 2).

## 6 Effect of gut microbiota on the efficacy of anti-tumor drugs

Studies have demonstrated that gut microbiota affects the efficacy of immune checkpoint inhibitors. The antimicrobial ability and efficacy of immune checkpoint inhibitors were significantly increased in mice colonized bya mixture of 11 strains compared with germ-free mice (Tanoue et al., 2019). In addition, healthy gut microbiota activates T cells through antigen presentation by APCs and promotes the expression of programmed cell death 1 (PD-1) and CTLA4 in these cells. This prevents immune damage caused by overactivation of T cells (Probst et al., 2005; Fink et al., 2007; Wei S C et al., 2019). However, following the occurrence of CRC, PD-1 and CTLA4 are highly expressed on activated T lymphocytes. This inhibits the activity of T cells, leading to uncontrolled growth of tumor cells (Yang et al., 2021). Recent studies have shown that the gut microbiota may enhance the effectiveness of tumor immunotherapy by blocking PD-1 and CTLA4. For example, *B. fragilis* can promote the maturation of DC in tumor cells and induce the activation of Th1 cells. Moreover, they exert antitumor effects by blocking CTLA4 (Vétizou et al., 2015). Notably, gut microbes can induce the production of anti-CD47 antibodies by activating STING signaling and improve the efficacy of immunotherapy. The accumulation of bifidobacteria in the TME significantly improves the antitumor efficacy of anti-CD47 immunotherapy, which relies on STING signaling within DC and type I IFN (Wolter et al., 2021). Bifidobacteria enhance the activation of CD8^+^ T cells and promote the aggregation of DC in the TME, resulting in a synergistic antitumor effect with anti-PD-L1 (Sivan et al., 2015). The intestinal flora also affects the antitumor activity of chemotherapy drugs. In the TME, the gut microbiome can respond to treatment with cyclophosphamide and oxaliplatin, mediate immune activation and affect the antitumor activity of the drugs (Iida et al., 2013; Viaud et al., 2013). Intestinal microbial metabolites can enhance the cytotoxic effect of 5-fluorouracil on CRC cells (González-Sarrías et al., 2015). The study also found that *F. nucleatum* activates the autophagy pathway and promotes chemoresistance in CRC by targeting TLR4 and MyD88 innate immune signaling and specific miRNAs (Yu et al., 2017).

## 7 Potential mechanisms of TCM in the treatment of CRC

TCM plays an important role in the development of tumors by inhibiting tumor cell infiltration and metastasis, promoting the apoptosis of tumor cells, and regulating the TME (Hsiao and Liu, 2010). The gut microbiota is one of the key factors for the efficacy of TCM. TCM and its active ingredients can regulate the composition of the intestinal microbiome, which in turn affects the remodeling of the TME and plays a role in the treatment of CRC (Table 1). Another study found that combination therapy with Gegen Qinlian decoction and anti-mouse PD-1 effectively inhibited the growth of CT26 tumors in xenograft models. It also affected the components of the gut microbiome (enriched for *s__Bacteroides_acidifaciens and s__uncultured_organism__g__norank_f__Bacteroidales_S24-7_group*), significantly increased the proportion of CD8^+^ T cells in peripheral blood and tumor tissues, downregulated PD-1, and increased the levels of IFN-γ and IL-2 (Lv et al., 2019). According to the ancient TCM book *Typhoid Fever*, Wu Mei Wan is commonly prescribed for the treatment of colitis. Studies have found that Wu Mei Wan can inhibit the transformation of Colitis-associated CRC by regulating the structure of the intestinal microbiome (i.e., decreased *Bacteroidetes* and increased *Firmicutes* at the phylum level, while decreasing *bacteroidales_s24-7_group* and increasing the number of Lachnospiraceae at the family level), restoring the balance of “tumor-progenic bacteria” and “tumor-suppressing bacteria” in the body, and downregulating the NF-κB/IL-6/STAT3 pathway. These effects prevent the occurrence of colitis-associated CRC (Jiang et al., 2020). Saponins extracted from *Gynostemma pentaphyllum* together with polysaccharides extracted from *Ganoderma lucidum* can significantly improve intestinal inflammation in Apc^Min/+^ mice, restore the intestinal mucosal barrier, shift colonic M1 to M2 macrophages, revert the E-cadherin/N-cadherin ratio, downregulate the expression of cancer-causing signaling molecules, and increase the number of bacteria producing SCFA, which play a role in preventing CRC (2019et al., 2019). In a mouse model of colitis-associated CRC induced by azoxymethane and dextran sulphate sodium, isoliquiritigenin (a flavonoid extracted from licorice) reduced the incidence of tumors. Isoliquiritigenin also increases the levels of probiotics, particularly in butyrate-producing bacteria (*Butyricicoccus, Clostridium, and Ruminococcus*), whereas it reduces the abundance of opportunistic pathogens (*Escherichia* and *Enterococcus*) (Wu et al., 2016). Resveratrol can affect tumor-infiltrating immune cells (i.e., increase the number of Treg and CD4^+^IL-10^+^ cells, and reduce that of inflammatory Th1 and Th17 cells), attenuate inflammation-driven CRC in mouse models by regulating the gut microbiota (Alrafas et al., 2020). Yi-Yi-Fu-Zi-Bai-Jiang-San can inhibit the growth of CRC cells by regulating the composition of the gut microbiome and affecting intestinal Treg cells (Sui et al., 2020). Parthenolide can exert anti-inflammatory and antitumor effects in mouse colon tissue through regulating the balance of Treg/Th17 cells and downregulating the levels of inflammatory factors (e.g., TNF-N, IL-1β, and IL-17A) (Liu et al., 2020). Treatment of CRC cells (SW620 and HT29) with glycyrrhizic acid accelerated apoptosis, reduced cell viability, and significantly reduced the protein levels of the colorectal oncogene sirtuin 3 (SIRT3) in a dose-dependent manner, thus exhibiting anti-cancer activity (Zuo et al., 2022). The naturally derived compound baicalein is a potent NF-κB inhibitor with antitumor effects on CRC. *In vitro* experiments have demonstrated that baicalein triggers apoptosis in CRC through the TLR4/NF-κB signaling pathway, inhibits cell migration, and improves the tumor immunosuppressive environment. This is achieved by downregulating the expression of PD-L1 and the proportion of MDSC, and increasing the percentage of CD4^+^ and CD8^+^ T cells. These effects improve antitumor immunity and significantly reduce tumor growth (Song et al., 2022).

**TABLE 1:** Potential mechanisms of TCM in the treatment of CRC.

TCM	Mechanisms of treatment of CRC	Effects on gut microbiota	References
Gegen Qinlian decoction (GQD)	Increases the proportion of CD8^+^ T cells, downregulates PD-1 while increasing the levels of IFN-γ and IL-2	enriches for *s__Bacteroides_acidifaciens and s__uncultured_organism_g__norank_f__Bacteroidales_S24-7_group*	Lv et al. (2019)
Wu Mei Wan (WMW)	Downregulates the NF-κB/IL-6/STAT3 pathway, thereby inhibiting the transformation of inflammatory cancers	decreases Bacteroidetes and increases Firmicutes at the phylum level, while decreasing *bacteroidales_s24-7_group* and increasing the number of Lachnospiraceae at the family level	Jiang et al. (2020)
GLP with GpS	Improves intestinal inflammation in Apc^Min/+^ mice, promotes colonic M2 macrophage polarization, restores E-cadherin/N-cadherin ratio, downregulates oncogenic signaling molecules	promots short-chain fatty acids (SCFAs)-producing bacteria and abridges sulfate-reducing bacteria	Khan et al. (2019)
Isoliquiritigenin (ISL)	Increases the level of probiotics, reduces the abundance of opportunistic pathogens (*Escherichia* and *enterococcus*)	increases the levels of probiotics, particularly in butyrate-producing bacteria (*Butyricicoccus*, *Clostridium*, and *Ruminococcus*), whereas it reduces the abundance of opportunistic pathogens (*Escherichia and Enterococcus*)	Wu et al. (2016)
Yi-Yi-Fu-Zi-Bai-Jiang-San (YYFZBJ)	Affects the number of Treg cells in the intestine	regulates animal’s natural gut flora, including *Bacteroides fragilis*, Lachnospiraceae and so on	Sui et al. (2020)
Parthenolide (PTL)	Regulates the balance of Treg/Th17 and downregulates the levels of inflammatory factors such as TNF-α, IL-1β, IL-17A	affects the abundance of *Alloprevotella* and *Bacteroides*	Liu et al. (2020)
Resveratrol	Increases Tregs and CD4+IL10+cells, reduces the number of inflammatory Th1 and Th17 cells	alters the gut microbiome and short chain fatty acid (SCFA), with modest increases in n-butyric acid and a potential butyrate precursor isobutyric acid	Alrafas et al. (2020)
Glycyrrhizic acid (GA)	Reduces the viability of colorectal cancer cells accelerate their apoptosis and reduces the protein levels of SIRT3		Zuo et al. (2022)
Baicalein (BA)	A potent NF-κB inhibitor, triggers apoptosis of CRC cells through the TLR4/NF-κB signaling pathway, inhibits their migration, downregulates PD-L1 expression and myeloid-derived suppressor cell (MDSC) ratio, and upregulates CD4^+^ and CD8^+^T cell ratios		Song et al. (2022)

GpS, Saponins from Gynostemma pentaphyllum; GLP, polysaccharides from Ganoderma lucidum.

## 8 Others

In addition to TCM, the use of probiotics and fecal microbial transplantation (FMT) has exhibited potential prospect of application in prevention and therapy of CRC by affecting TME. It has been found that probiotics can prevent and treat CRC by regulating the composition of intestinal flora (such as increasing the abundance of butyrate-producing bacteria), inhibiting the activity of pathogenic bacteria, regulating host immunity (such as promoting T cell differentiation and DC maturation), and improving intestinal barrier function (Raman et al., 2013; Cai et al., 2016). In recent years, studies have shown that FMT can enhance the antitumor effect of immune checkpoint inhibitors and overcome the resistance of immunotherapy (Furusawa et al., 2013; Matson et al., 2018; Routy et al., 2018). However, clinical studies have found that FMT may cause infectious adverse events such as bacteremia, which makes its long-term safety worrisome (DeFilipp et al., 2019).

## 9 Conclusion

The gut microbiome plays a vital role in regulating tumor progression and prognosis in CRC through its effects on the TME. Moreover, crosstalk between the intestinal immune system and the gut microbiome can affect the progression of cancer. TCM offers unique advantages in the treatment and prevention of diseases. The gut microbiome exhibits a strong impact on the efficacy of TCM through the conversion of drugs into bioactive metabolites that are easily absorbed by the body, thus improving the efficacy of drugs. Studies have revealed that TCM can indirectly reshape the TME by regulation of gut mircobiota. This article briefly discusses the effects of gut microbiome and its metabolites on inflammation and various cells in the TME. In addition, potential mechanisms concerning TCM in the treatment of CRC are summarized. Nevertheless, the present review does not clearly indicate the type of patients who may benefit from combination treatment with TCM and antitumor drugs. Although the results of researches concerning TCM in treating CRC are encouraging, there is a lack of direct clinical studies that confirm TCM can exert antitumor effects by regulating the intestinal flora. Hence, further investigation on gut mircobiota is warranted to identify the key targets and signal pathways of TCM *in vivo*. In addition, the complex composition and various therapeutic effects of TCM require individualized treatment regimens, making it difficult to conduct large-scale clinical trials. The active ingredients of some TCM may have certain liver and kidney toxicity, which also limits the application scope of TCM.

## References

[B1] Alrafas H. R. Busbee P. B. Chitrala K. N. Nagarkatti M. Nagarkatti P. (2020). Alterations in the gut microbiome and suppression of histone deacetylases by resveratrol are associated with attenuation of colonic inflammation and protection against colorectal cancer. J. Clin. Med. 9, 1796. 10.3390/jcm9061796 32526927 PMC7355848

[B2] Arpaia N. Campbell C. Fan X. Dikiy S. Van Der Veeken J. Deroos P. (2013). Metabolites produced by commensal bacteria promote peripheral regulatory T-cell generation. Nature 504, 451–455. 10.1038/nature12726 24226773 PMC3869884

[B3] Baidoun F. Elshiwy K. Elkeraie Y. Merjaneh Z. Khoudari G. Sarmini M. T. (2021). Colorectal cancer epidemiology: Recent trends and impact on outcomes. Curr. Drug Targets 22, 998–1009. 10.2174/1389450121999201117115717 33208072

[B4] Bashir A. Miskeen A. Y. Hazari Y. M. Asrafuzzaman S. Fazili K. M. (2016). Fusobacterium nucleatum, inflammation, and immunity: The fire within human gut. Tumour Biol. 37, 2805–2810. 10.1007/s13277-015-4724-0 26718210

[B5] Boleij A. Tjalsma H. (2012). Gut bacteria in health and disease: A survey on the interface between intestinal microbiology and colorectal cancer. Biol. Rev. Camb Philos. Soc. 87, 701–730. 10.1111/j.1469-185X.2012.00218.x 22296522

[B6] Böttcher J. P. Bonavita E. Chakravarty P. Blees H. Cabeza-Cabrerizo M. Sammicheli S. (2018). NK cells stimulate recruitment of cDC1 into the tumor microenvironment promoting cancer immune control. Cell 172, 1022–1037. 10.1016/j.cell.2018.01.004 29429633 PMC5847168

[B7] Cai S. Kandasamy M. Rahmat J. N. Tham S. M. Bay B. H. Lee Y. K. (2016). Lactobacillus rhamnosus GG activation of dendritic cells and neutrophils depends on the dose and time of exposure. J. Immunol. Res. 2016, 7402760. 10.1155/2016/7402760 27525288 PMC4971325

[B8] Campbell C. Mckenney P. T. Konstantinovsky D. Isaeva O. I. Schizas M. Verter J. (2020). Bacterial metabolism of bile acids promotes generation of peripheral regulatory T cells. Nature 581, 475–479. 10.1038/s41586-020-2193-0 32461639 PMC7540721

[B9] Canavan C. Abrams K. R. Mayberry J. (2006). Meta-analysis: Colorectal and small bowel cancer risk in patients with crohn's disease. Aliment. Pharmacol. Ther. 23, 1097–1104. 10.1111/j.1365-2036.2006.02854.x 16611269

[B10] Carmeliet P. (2005). VEGF as a key mediator of angiogenesis in cancer. Oncology 69 (3), 4–10. 10.1159/000088478 16301830

[B11] Cervelli M. Pietropaoli S. Signore F. Amendola R. Mariottini P. (2014). Polyamines metabolism and breast cancer: State of the art and perspectives. Breast Cancer Res. Treat. 148, 233–248. 10.1007/s10549-014-3156-7 25292420

[B12] Cheng Y. Ling Z. Li L. (2020). The intestinal microbiota and colorectal cancer. Front. Immunol. 11, 615056. 10.3389/fimmu.2020.615056 33329610 PMC7734048

[B13] Clay S. L. Fonseca-Pereira D. Garrett W. S. (2022). Colorectal cancer: The facts in the case of the microbiota. J. Clin. Invest. 132, e155101. 10.1172/JCI155101 35166235 PMC8843708

[B14] Coni S. Di Magno L. Serrao S. M. Kanamori Y. Agostinelli E. Canettieri G. (2019). Polyamine metabolism as a therapeutic target inHedgehog-driven basal cell carcinomaand medulloblastoma. Cells 8. 10.3390/cells8020150 PMC6406590 30754726

[B15] Davie J. R. (2003). Inhibition of histone deacetylase activity by butyrate. J. Nutr. 133, 2485S–2493s. 10.1093/jn/133.7.2485S 12840228

[B16] Defilipp Z. Bloom P. P. Torres Soto M. Mansour M. K. Sater M. R. A. Huntley M. H. (2019). Drug-Resistant *E. coli* bacteremia transmitted by fecal microbiota transplant. N. Engl. J. Med. 381, 2043–2050. 10.1056/NEJMoa1910437 31665575

[B17] Eaden J. A. Abrams K. R. Mayberry J. F. (2001). The risk of colorectal cancer in ulcerative colitis: A meta-analysis. Gut 48, 526–535. 10.1136/gut.48.4.526 11247898 PMC1728259

[B18] Erdman S. E. Rao V. P. Poutahidis T. Rogers A. B. Taylor C. L. Jackson E. A. (2009). Nitric oxide and TNF-alpha trigger colonic inflammation and carcinogenesis in Helicobacter hepaticus-infected, Rag2-deficient mice. Proc. Natl. Acad. Sci. U. S. A. 106, 1027–1032. 10.1073/pnas.0812347106 19164562 PMC2633549

[B19] Farraye F. A. Odze R. D. Eaden J. Itzkowitz S. H. Mccabe R. P. Dassopoulos T. (2010). AGA medical position statement on the diagnosis and management of colorectal neoplasia in inflammatory bowel disease. Gastroenterology 138, 738–745. 10.1053/j.gastro.2009.12.037 20141808

[B20] Fink L. N. Zeuthen L. H. Ferlazzo G. Frøkiaer H. (2007). Human antigen-presenting cells respond differently to gut-derived probiotic bacteria but mediate similar strain-dependent NK and T cell activation. FEMS Immunol. Med. Microbiol. 51, 535–546. 10.1111/j.1574-695X.2007.00333.x 17903206

[B21] Furusawa Y. Obata Y. Fukuda S. Endo T. A. Nakato G. Takahashi D. (2013). Commensal microbe-derived butyrate induces the differentiation of colonic regulatory T cells. Nature 504, 446–450. 10.1038/nature12721 24226770

[B22] Gabrilovich D. I. Ostrand-Rosenberg S. Bronte V. (2012). Coordinated regulation of myeloid cells by tumours. Nat. Rev. Immunol. 12, 253–268. 10.1038/nri3175 22437938 PMC3587148

[B23] Gao Y. Bi D. Xie R. Li M. Guo J. Liu H. (2021). Fusobacterium nucleatum enhances the efficacy of PD-L1 blockade in colorectal cancer. Signal Transduct. Target Ther. 6, 398. 10.1038/s41392-021-00795-x 34795206 PMC8602417

[B24] González-Sarrías A. Tomé-Carneiro J. Bellesia A. Tomás-Barberán F. A. Espín J. C. (2015). The ellagic acid-derived gut microbiota metabolite, urolithin A, potentiates the anticancer effects of 5-fluorouracil chemotherapy on human colon cancer cells. Food Funct. 6, 1460–1469. 10.1039/c5fo00120j 25857357

[B25] Gordon S. (2003). Alternative activation of macrophages. Nat. Rev. Immunol. 3, 23–35. 10.1038/nri978 12511873

[B26] Grivennikov S. I. Wang K. Mucida D. Stewart C. A. Schnabl B. Jauch D. (2012). Adenoma-linked barrier defects and microbial products drive IL-23/IL-17-mediated tumour growth. Nature 491, 254–258. 10.1038/nature11465 23034650 PMC3601659

[B27] Gur C. Ibrahim Y. Isaacson B. Yamin R. Abed J. Gamliel M. (2015). Binding of the Fap2 protein of Fusobacterium nucleatum to human inhibitory receptor TIGIT protects tumors from immune cell attack. Immunity 42, 344–355. 10.1016/j.immuni.2015.01.010 25680274 PMC4361732

[B28] Hanus M. Parada-Venegas D. Landskron G. Wielandt A. M. Hurtado C. Alvarez K. (2021). Immune system, microbiota, and microbial metabolites: The unresolved triad in colorectal cancer microenvironment. Front. Immunol. 12, 612826. 10.3389/fimmu.2021.612826 33841394 PMC8033001

[B29] Hardbower D. M. Asim M. Luis P. B. Singh K. Barry D. P. Yang C. (2017). Ornithine decarboxylase regulates M1 macrophage activation and mucosal inflammation via histone modifications. Proc. Natl. Acad. Sci. U. S. A. 114, E751–e760. 10.1073/pnas.1614958114 28096401 PMC5293075

[B30] Haskó G. Kuhel D. G. Marton A. Nemeth Z. H. Deitch E. A. Szabó C. (2000). Spermine differentially regulates the production of interleukin-12 p40 and interleukin-10 and suppresses the release of the T helper 1 cytokine interferon-gamma. Shock 14, 144–149. 10.1097/00024382-200014020-00012 10947158

[B31] Hayase E. Jenq R. R. (2021). Role of the intestinal microbiome and microbial-derived metabolites in immune checkpoint blockade immunotherapy of cancer. Genome Med. 13, 107. 10.1186/s13073-021-00923-w 34162429 PMC8220726

[B32] Hesterberg R. S. Cleveland J. L. Epling-Burnette P. K. (2018). Role of polyamines in immune cell functions. Med. Sci. (Basel) 6, 22. 10.3390/medsci6010022 29517999 PMC5872179

[B33] Hibberd A. A. Lyra A. Ouwehand A. C. Rolny P. Lindegren H. Cedgård L. (2017). Intestinal microbiota is altered in patients with colon cancer and modified by probiotic intervention. BMJ Open Gastroenterol. 4, e000145. 10.1136/bmjgast-2017-000145 PMC5609083 28944067

[B34] Hsiao W. L. Liu L. (2010). The role of traditional Chinese herbal medicines in cancer therapy--from TCM theory to mechanistic insights. Planta Med. 76, 1118–1131. 10.1055/s-0030-1250186 20635308

[B35] Huang J. Sun R. Qi X. Liu L. Yang Y. Sun B. (2019). Effect of autophagy on expression of neutrophil programmed death ligand-1 in mice with sepsis. Zhonghua Wei Zhong Bing Ji Jiu Yi Xue 31, 1091–1096. 10.3760/cma.j.issn.2095-4352.2019.09.007 31657331

[B36] Iida N. Dzutsev A. Stewart C. A. Smith L. Bouladoux N. Weingarten R. A. (2013). Commensal bacteria control cancer response to therapy by modulating the tumor microenvironment. Science 342, 967–970. 10.1126/science.1240527 24264989 PMC6709532

[B37] Jain T. Sharma P. Are A. C. Vickers S. M. Dudeja V. (2021). New insights into the cancer-microbiome-immune Axis: Decrypting a decade of discoveries. Front. Immunol. 12, 622064. 10.3389/fimmu.2021.622064 33708214 PMC7940198

[B38] Jiang F. Liu M. Wang H. Shi G. Chen B. Chen T. (2020). Wu Mei Wan attenuates CAC by regulating gut microbiota and the NF-kB/IL6-STAT3 signaling pathway. Biomed. Pharmacother. 125, 109982. 10.1016/j.biopha.2020.109982 32119646

[B39] Johnson C. H. Dejea C. M. Edler D. Hoang L. T. Santidrian A. F. Felding B. H. (2015). Metabolism links bacterial biofilms and colon carcinogenesis. Cell Metab. 21, 891–897. 10.1016/j.cmet.2015.04.011 25959674 PMC4456201

[B40] Khan I. Huang G. Li X. A. Liao W. Leong W. K. Xia W. (2019). Mushroom polysaccharides and jiaogulan saponins exert cancer preventive effects by shaping the gut microbiota and microenvironment in Apc(Min/+) mice. Pharmacol. Res. 148, 104448. 10.1016/j.phrs.2019.104448 31499195

[B41] Kostic A. D. Chun E. Robertson L. Glickman J. N. Gallini C. A. Michaud M. (2013). Fusobacterium nucleatum potentiates intestinal tumorigenesis and modulates the tumor-immune microenvironment. Cell Host Microbe 14, 207–215. 10.1016/j.chom.2013.07.007 23954159 PMC3772512

[B42] Latour Y. L. Gobert A. P. Wilson K. T. (2020). The role of polyamines in the regulation of macrophage polarization and function. Amino Acids 52, 151–160. 10.1007/s00726-019-02719-0 31016375 PMC6812587

[B43] Lee T. C. Huang Y. C. Lu Y. Z. Yeh Y. C. Yu L. C. (2018). Hypoxia-induced intestinal barrier changes in balloon-assisted enteroscopy. J. Physiol. 596, 3411–3424. 10.1113/JP275277 29178568 PMC6068115

[B44] Li Q. Ma L. Shen S. Guo Y. Cao Q. Cai X. (2019a). Intestinal dysbacteriosis-induced IL-25 promotes development of HCC via alternative activation of macrophages in tumor microenvironment. J. Exp. Clin. Cancer Res. 38, 303. 10.1186/s13046-019-1271-3 31296243 PMC6625119

[B45] Li R. Zhou R. Wang H. Li W. Pan M. Yao X. (2019b). Gut microbiota-stimulated cathepsin K secretion mediates TLR4-dependent M2 macrophage polarization and promotes tumor metastasis in colorectal cancer. Cell Death Differ. 26, 2447–2463. 10.1038/s41418-019-0312-y 30850734 PMC6889446

[B46] Li Y. Kundu P. Seow S. W. De Matos C. T. Aronsson L. Chin K. C. (2012). Gut microbiota accelerate tumor growth via c-jun and STAT3 phosphorylation in APCMin/+ mice. Carcinogenesis 33, 1231–1238. 10.1093/carcin/bgs137 22461519

[B47] Liu Y. J. Tang B. Wang F. C. Tang L. Lei Y. Y. Luo Y. (2020). Parthenolide ameliorates colon inflammation through regulating Treg/Th17 balance in a gut microbiota-dependent manner. Theranostics 10, 5225–5241. 10.7150/thno.43716 32373209 PMC7196297

[B48] Long X. Wong C. C. Tong L. Chu E. S. H. Ho Szeto C. Go M. Y. Y. (2019). Peptostreptococcus anaerobius promotes colorectal carcinogenesis and modulates tumour immunity. Nat. Microbiol. 4, 2319–2330. 10.1038/s41564-019-0541-3 31501538

[B49] Lucas C. Barnich N. Nguyen H. T. T. (2017). Microbiota, inflammation and colorectal cancer. Int. J. Mol. Sci. 18, 1310. 10.3390/ijms18061310 28632155 PMC5486131

[B50] Lv J. Jia Y. Li J. Kuai W. Li Y. Guo F. (2019). Gegen Qinlian decoction enhances the effect of PD-1 blockade in colorectal cancer with microsatellite stability by remodelling the gut microbiota and the tumour microenvironment. Cell Death Dis. 10, 415. 10.1038/s41419-019-1638-6 31138779 PMC6538740

[B51] Mager L. F. Burkhard R. Pett N. Cooke N. C. A. Brown K. Ramay H. (2020). Microbiome-derived inosine modulates response to checkpoint inhibitor immunotherapy. Science 369, 1481–1489. 10.1126/science.abc3421 32792462

[B52] Matson V. Fessler J. Bao R. Chongsuwat T. Zha Y. Alegre M. L. (2018). The commensal microbiome is associated with anti-PD-1 efficacy in metastatic melanoma patients. Science 359, 104–108. 10.1126/science.aao3290 29302014 PMC6707353

[B53] Maynard C. L. Elson C. O. Hatton R. D. Weaver C. T. (2012). Reciprocal interactions of the intestinal microbiota and immune system. Nature 489, 231–241. 10.1038/nature11551 22972296 PMC4492337

[B54] Mcdermott A. J. Huffnagle G. B. (2014). The microbiome and regulation of mucosal immunity. Immunology 142, 24–31. 10.1111/imm.12231 24329495 PMC3992044

[B55] Milovic V. Turchanowa L. (2003). Polyamines and colon cancer. Biochem. Soc. Trans. 31, 381–383. 10.1042/bst0310381 12653643

[B56] Mima K. Sukawa Y. Nishihara R. Qian Z. R. Yamauchi M. Inamura K. (2015). Fusobacterium nucleatum and T cells in colorectal carcinoma. JAMA Oncol. 1, 653–661. 10.1001/jamaoncol.2015.1377 26181352 PMC4537376

[B57] Overacre-Delgoffe A. E. Bumgarner H. J. Cillo A. R. Burr A. H. P. Tometich J. T. Bhattacharjee A. (2021). Microbiota-specific T follicular helper cells drive tertiary lymphoid structures and anti-tumor immunity against colorectal cancer. Immunity 54, 2812–2824. 10.1016/j.immuni.2021.11.003 34861182 PMC8865366

[B58] Poggi A. Benelli R. Venè R. Costa D. Ferrari N. Tosetti F. (2019). Human gut-associated natural killer cells in health and disease. Front. Immunol. 10, 961. 10.3389/fimmu.2019.00961 31130953 PMC6509241

[B59] Pouysségur J. Dayan F. Mazure N. M. (2006). Hypoxia signalling in cancer and approaches to enforce tumour regression. Nature 441, 437–443. 10.1038/nature04871 16724055

[B60] Probst H. C. Mccoy K. Okazaki T. Honjo T. Van Den Broek M. (2005). Resting dendritic cells induce peripheral CD8^+^ T cell tolerance through PD-1 and CTLA-4. Nat. Immunol. 6, 280–286. 10.1038/ni1165 15685176

[B61] Qi F. Zhao L. Zhou A. Zhang B. Li A. Wang Z. (2015). The advantages of using traditional Chinese medicine as an adjunctive therapy in the whole course of cancer treatment instead of only terminal stage of cancer. Biosci. Trends 9, 16–34. 10.5582/bst.2015.01019 25787906

[B62] Qiu Q. Lin Y. Ma Y. Li X. Liang J. Chen Z. (2020). Exploring the emerging role of the gut microbiota and tumor microenvironment in cancer immunotherapy. Front. Immunol. 11, 612202. 10.3389/fimmu.2020.612202 33488618 PMC7817884

[B63] Raber P. L. Thevenot P. Sierra R. Wyczechowska D. Halle D. Ramirez M. E. (2014). Subpopulations of myeloid-derived suppressor cells impair T cell responses through independent nitric oxide-related pathways. Int. J. Cancer 134, 2853–2864. 10.1002/ijc.28622 24259296 PMC3980009

[B64] Raman M. Ambalam P. Kondepudi K. K. Pithva S. Kothari C. Patel A. T. (2013). Potential of probiotics, prebiotics and synbiotics for management of colorectal cancer. Gut Microbes 4, 181–192. 10.4161/gmic.23919 23511582 PMC3669163

[B65] Ramos-Molina B. Queipo-Ortuño M. I. Lambertos A. Tinahones F. J. Peñafiel R. (2019). Dietary and gut microbiota polyamines in obesity- and age-related diseases. Front. Nutr. 6, 24. 10.3389/fnut.2019.00024 30923709 PMC6426781

[B66] Ren L. Ye J. Zhao B. Sun J. Cao P. Yang Y. (2021). The role of intestinal microbiota in colorectal cancer. Front. Pharmacol. 12, 674807. 10.3389/fphar.2021.674807 33959032 PMC8093878

[B67] Routy B. Le Chatelier E. Derosa L. Duong C. P. M. Alou M. T. Daillère R. (2018). Gut microbiome influences efficacy of PD-1-based immunotherapy against epithelial tumors. Science 359, 91–97. 10.1126/science.aan3706 29097494

[B68] Schmitt M. Greten F. R. (2021). The inflammatory pathogenesis of colorectal cancer. Nat. Rev. Immunol. 21, 653–667. 10.1038/s41577-021-00534-x 33911231

[B69] Schreiber R. D. Old L. J. Smyth M. J. (2011). Cancer immunoediting: Integrating immunity's roles in cancer suppression and promotion. Science 331, 1565–1570. 10.1126/science.1203486 21436444

[B70] Sepich-Poore G. D. Zitvogel L. Straussman R. Hasty J. Wargo J. A. Knight R. (2021). The microbiome and human cancer. Science 371, eabc4552. 10.1126/science.abc4552 33766858 PMC8767999

[B71] Shang K. Bai Y. P. Wang C. Wang Z. Gu H. Y. Du X. (2012). Crucial involvement of tumor-associated neutrophils in the regulation of chronic colitis-associated carcinogenesis in mice. PLoS One 7, e51848. 10.1371/journal.pone.0051848 23272179 PMC3525572

[B72] Shenker B. J. Datar S. (1995). Fusobacterium nucleatum inhibits human T-cell activation by arresting cells in the mid-G1 phase of the cell cycle. Infect. Immun. 63, 4830–4836. 10.1128/IAI.63.12.4830-4836.1995 7591143 PMC173692

[B73] Shi N. Li N. Duan X. Niu H. (2017). Interaction between the gut microbiome and mucosal immune system. Mil. Med. Res. 4, 14. 10.1186/s40779-017-0122-9 28465831 PMC5408367

[B74] Shi Y. Zheng W. Yang K. Harris K. G. Ni K. Xue L. (2020). Intratumoral accumulation of gut microbiota facilitates CD47-based immunotherapy via STING signaling. J. Exp. Med. 217, e20192282. 10.1084/jem.20192282 32142585 PMC7201921

[B75] Singh N. Gurav A. Sivaprakasam S. Brady E. Padia R. Shi H. (2014). Activation of Gpr109a, receptor for niacin and the commensal metabolite butyrate, suppresses colonic inflammation and carcinogenesis. Immunity 40, 128–139. 10.1016/j.immuni.2013.12.007 24412617 PMC4305274

[B76] Sivan A. Corrales L. Hubert N. Williams J. B. Aquino-Michaels K. Earley Z. M. (2015). Commensal Bifidobacterium promotes antitumor immunity and facilitates anti-PD-L1 efficacy. Science 350, 1084–1089. 10.1126/science.aac4255 26541606 PMC4873287

[B77] Smith P. M. Howitt M. R. Panikov N. Michaud M. Gallini C. A. Bohlooly Y. M. (2013). The microbial metabolites, short-chain fatty acids, regulate colonic Treg cell homeostasis. Science 341, 569–573. 10.1126/science.1241165 23828891 PMC3807819

[B78] Soda K. (2011). The mechanisms by which polyamines accelerate tumor spread. J. Exp. Clin. Cancer Res. 30, 95. 10.1186/1756-9966-30-95 21988863 PMC3206444

[B79] Sommer F. Bäckhed F. (2013). The gut microbiota--masters of host development and physiology. Nat. Rev. Microbiol. 11, 227–238. 10.1038/nrmicro2974 23435359

[B80] Song L. Zhu S. Liu C. Zhang Q. Liang X. (2022). Baicalin triggers apoptosis, inhibits migration, and enhances anti-tumor immunity in colorectal cancer via TLR4/NF-κB signaling pathway. J. Food Biochem. 46, e13703. 10.1111/jfbc.13703 33742464

[B81] Song X. Gao H. Lin Y. Yao Y. Zhu S. Wang J. (2014). Alterations in the microbiota drive interleukin-17C production from intestinal epithelial cells to promote tumorigenesis. Immunity 40, 140–152. 10.1016/j.immuni.2013.11.018 24412611

[B82] Sui H. Zhang L. Gu K. Chai N. Ji Q. Zhou L. (2020). YYFZBJS ameliorates colorectal cancer progression in Apc(Min/+) mice by remodeling gut microbiota and inhibiting regulatory T-cell generation. Cell Commun. Signal 18, 113. 10.1186/s12964-020-00596-9 32677955 PMC7367414

[B83] Suzuki T. (2020). Regulation of the intestinal barrier by nutrients: The role of tight junctions. Anim. Sci. J. 91, e13357. 10.1111/asj.13357 32219956 PMC7187240

[B84] Takiishi T. Fenero C. I. M. Câmara N. O. S. (2017). Intestinal barrier and gut microbiota: Shaping our immune responses throughout life. Tissue Barriers 5, e1373208. 10.1080/21688370.2017.1373208 28956703 PMC5788425

[B85] Tang B. Wang K. Jia Y. P. Zhu P. Fang Y. Zhang Z. J. (2016). Fusobacterium nucleatum-induced impairment of autophagic flux enhances the expression of proinflammatory cytokines via ROS in caco-2 cells. PLoS One 11, e0165701. 10.1371/journal.pone.0165701 27828984 PMC5102440

[B86] Tanoue T. Morita S. Plichta D. R. Skelly A. N. Suda W. Sugiura Y. (2019). A defined commensal consortium elicits CD8 T cells and anti-cancer immunity. Nature 565, 600–605. 10.1038/s41586-019-0878-z 30675064

[B87] Thiele Orberg E. Fan H. Tam A. J. Dejea C. M. Destefano Shields C. E. Wu S. (2017). The myeloid immune signature of enterotoxigenic Bacteroides fragilis-induced murine colon tumorigenesis. Mucosal Immunol. 10, 421–433. 10.1038/mi.2016.53 27301879 PMC5159334

[B88] Thornton R. D. Lane P. Borghaei R. C. Pease E. A. Caro J. Mochan E. (2000). Interleukin 1 induces hypoxia-inducible factor 1 in human gingival and synovial fibroblasts. Biochem. J. 350, 307–312. 10.1042/bj3500307 10926858 PMC1221256

[B89] Topping D. L. Clifton P. M. (2001). Short-chain fatty acids and human colonic function: Roles of resistant starch and nonstarch polysaccharides. Physiol. Rev. 81, 1031–1064. 10.1152/physrev.2001.81.3.1031 11427691

[B90] Triner D. Devenport S. N. Ramakrishnan S. K. Ma X. Frieler R. A. Greenson J. K. (2019). Neutrophils restrict tumor-associated microbiota to reduce growth and invasion of colon tumors in mice. Gastroenterology 156, 1467–1482. 10.1053/j.gastro.2018.12.003 30550822 PMC6441634

[B91] Tsujinaka S. Soda K. Kano Y. Konishi F. (2011). Spermine accelerates hypoxia-initiated cancer cell migration. Int. J. Oncol. 38, 305–312. 10.3892/ijo.2010.849 21132262

[B92] Vétizou M. Pitt J. M. Daillère R. Lepage P. Waldschmitt N. Flament C. (2015). Anticancer immunotherapy by CTLA-4 blockade relies on the gut microbiota. Science 350, 1079–1084. 10.1126/science.aad1329 26541610 PMC4721659

[B93] Viaud S. Saccheri F. Mignot G. Yamazaki T. Daillère R. Hannani D. (2013). The intestinal microbiota modulates the anticancer immune effects of cyclophosphamide. Science 342, 971–976. 10.1126/science.1240537 24264990 PMC4048947

[B94] Wang H. Tian T. Zhang J. (2021). Tumor-associated macrophages (TAMs) in colorectal cancer (CRC): From mechanism to therapy and prognosis. Int. J. Mol. Sci. 22, 8470. 10.3390/ijms22168470 34445193 PMC8395168

[B95] Wang X. Huycke M. M. (2007). Extracellular superoxide production by *Enterococcus faecalis* promotes chromosomal instability in mammalian cells. Gastroenterology 132, 551–561. 10.1053/j.gastro.2006.11.040 17258726

[B96] Wang Y. Wang M. Wu H. X. Xu R. H. (2021). Advancing to the era of cancer immunotherapy. Cancer Commun. (Lond) 41, 803–829. 10.1002/cac2.12178 34165252 PMC8441060

[B97] Wculek S. K. Amores-Iniesta J. Conde-Garrosa R. Khouili S. C. Melero I. Sancho D. (2019). Effective cancer immunotherapy by natural mouse conventional type-1 dendritic cells bearing dead tumor antigen. J. Immunother. Cancer 7, 100. 10.1186/s40425-019-0565-5 30961656 PMC6454603

[B98] Wei C. Yang C. Wang S. Shi D. Zhang C. Lin X. (2019). Crosstalk between cancer cells and tumor associated macrophages is required for mesenchymal circulating tumor cell-mediated colorectal cancer metastasis. Mol. Cancer 18, 64. 10.1186/s12943-019-0976-4 30927925 PMC6441214

[B99] Wei S. C. Sharma R. Anang N. a. S. Levine J. H. Zhao Y. Mancuso J. J. (2019). Negative Co-stimulation constrains T cell differentiation by imposing boundaries on possible cell states. Immunity 50, 1084–1098. 10.1016/j.immuni.2019.03.004 30926234 PMC6664799

[B100] Westra J. Brouwer E. Bos R. Posthumus M. D. Doornbos-Van Der Meer B. Kallenberg C. G. (2007). Regulation of cytokine-induced HIF-1alpha expression in rheumatoid synovial fibroblasts. Ann. N. Y. Acad. Sci. 1108, 340–348. 10.1196/annals.1422.035 17893997

[B101] Wolter M. Grant E. T. Boudaud M. Steimle A. Pereira G. V. Martens E. C. (2021). Leveraging diet to engineer the gut microbiome. Nat. Rev. Gastroenterol. Hepatol. 18, 885–902. 10.1038/s41575-021-00512-7 34580480

[B102] Wu M. Wu Y. Deng B. Li J. Cao H. Qu Y. (2016). Isoliquiritigenin decreases the incidence of colitis-associated colorectal cancer by modulating the intestinal microbiota. Oncotarget 7, 85318–85331. 10.18632/oncotarget.13347 27863401 PMC5356739

[B103] Wu S. Rhee K. J. Zhang M. Franco A. Sears C. L. (2007). Bacteroides fragilis toxin stimulates intestinal epithelial cell shedding and gamma-secretase-dependent E-cadherin cleavage. J. Cell Sci. 120, 1944–1952. 10.1242/jcs.03455 17504810 PMC3056613

[B104] Xu C. Fan L. Lin Y. Shen W. Qi Y. Zhang Y. (2021). Fusobacterium nucleatum promotes colorectal cancer metastasis through miR-1322/CCL20 axis and M2 polarization. Gut Microbes 13, 1980347. 10.1080/19490976.2021.1980347 34632963 PMC8510564

[B105] Yang W. Yu T. Huang X. Bilotta A. J. Xu L. Lu Y. (2020). Intestinal microbiota-derived short-chain fatty acids regulation of immune cell IL-22 production and gut immunity. Nat. Commun. 11, 4457. 10.1038/s41467-020-18262-6 32901017 PMC7478978

[B106] Yang X. Guo Y. Chen C. Shao B. Zhao L. Zhou Q. (2021). Interaction between intestinal microbiota and tumour immunity in the tumour microenvironment. Immunology 164, 476–493. 10.1111/imm.13397 34322877 PMC8517597

[B107] Yang Y. Misra B. B. Liang L. Bi D. Weng W. Wu W. (2019). Integrated microbiome and metabolome analysis reveals a novel interplay between commensal bacteria and metabolites in colorectal cancer. Theranostics 9, 4101–4114. 10.7150/thno.35186 31281534 PMC6592169

[B108] Yu T. Guo F. Yu Y. Sun T. Ma D. Han J. (2017). Fusobacterium nucleatum promotes chemoresistance to colorectal cancer by modulating autophagy. Cell 170, 548–563. 10.1016/j.cell.2017.07.008 28753429 PMC5767127

[B109] Zagato E. Pozzi C. Bertocchi A. Schioppa T. Saccheri F. Guglietta S. (2020). Endogenous murine microbiota member Faecalibaculum rodentium and its human homologue protect from intestinal tumour growth. Nat. Microbiol. 5, 511–524. 10.1038/s41564-019-0649-5 31988379 PMC7048616

[B110] Zhang M. Caragine T. Wang H. Cohen P. S. Botchkina G. Soda K. (1997). Spermine inhibits proinflammatory cytokine synthesis in human mononuclear cells: A counterregulatory mechanism that restrains the immune response. J. Exp. Med. 185, 1759–1768. 10.1084/jem.185.10.1759 9151701 PMC2196317

[B111] Zhang Y. Lou Y. Wang J. Yu C. Shen W. (2020). Research status and molecular mechanism of the traditional Chinese medicine and antitumor therapy combined strategy based on tumor microenvironment. Front. Immunol. 11, 609705. 10.3389/fimmu.2020.609705 33552068 PMC7859437

[B112] Zhao H. He M. Zhang M. Sun Q. Zeng S. Chen L. (2021). Colorectal cancer, gut microbiota and traditional Chinese medicine: A systematic review. Am. J. Chin. Med. 49, 805–828. 10.1142/S0192415X21500385 33827382

[B113] Zuo L. Lu M. Zhou Q. Wei W. Wang Y. (2013). Butyrate suppresses proliferation and migration of RKO colon cancer cells though regulating endocan expression by MAPK signaling pathway. Food Chem. Toxicol. 62, 892–900. 10.1016/j.fct.2013.10.028 24416777

[B114] Zuo Z. He L. Duan X. Peng Z. Han J. (2022). Glycyrrhizic acid exhibits strong anticancer activity in colorectal cancer cells via SIRT3 inhibition. Bioengineered 13, 2720–2731. 10.1080/21655979.2021.2001925 34747319 PMC8974138

